# Interphase Surface Stability in Liquid-Liquid Membrane Contactors Based on Track-Etched Membranes

**DOI:** 10.3390/membranes11120949

**Published:** 2021-11-30

**Authors:** Stepan Bazhenov, Olga Kristavchuk, Margarita Kostyanaya, Anton Belogorlov, Ruslan Ashimov, Pavel Apel

**Affiliations:** 1 A.V. Topchiev Institute of Petrochemical Synthesis, Russian Academy of Sciences, 119991 Moscow, Russia; marille@ips.ac.ru (M.K.); aabelogorlov@ips.ac.ru (A.B.); ashimov999@gmail.com (R.A.); 2 Flerov Laboratory of Nuclear Reactions, Joint Institute for Nuclear Research, 141980 Dubna, Russia; kristavchuk@jinr.ru (O.K.); apel@jinr.ru (P.A.); 3 Molecular Physics Department, National Research Nuclear University Moscow Engineering Physics Institute, 115409 Moscow, Russia; 4 Research Institute for Graphite-Based Structural Materials “NIIgrafit” (JSC “NIIgrafit”), 111524 Moscow, Russia

**Keywords:** track-etched membrane, liquid–liquid membrane contactor, liquid–liquid displacement

## Abstract

A promising solution for the implementation of extraction processes is liquid–liquid membrane contactors. The transfer of the target component from one immiscible liquid to another is carried out inside membrane pores. For the first time, highly asymmetric track-etched membranes made of polyethylene terephthalate (PET) of the same thickness but with different pore diameters (12.5–19 nm on one side and hundreds of nanometers on the other side) were studied in the liquid–liquid membrane contactor. For analysis of the liquid–liquid interface stability, two systems widely diverging in the interfacial tension value were used: water–pentanol and water–hexadecane. The interface stability was investigated depending on the following process parameters: the porous structure, the location of the asymmetric membrane in the contactor, the velocities of liquids, and the pressure drop between them. It was shown that the stability of the interface increases with decreasing pore size. Furthermore, it is preferable to supply the aqueous phase from the side of the asymmetric membrane with the larger pore size. The asymmetry of the porous structure of the membrane makes it possible to increase the range of pressure drop values between the phases by at least two times (from 5 to 10 kPa), which does not lead to mutual dispersion of the liquids. The liquid–liquid contactor based on the asymmetric track-etched membranes allows for the extraction of impurities from the organic phase into the aqueous phase by using a 1% solution of acetone in hexadecane as an example.

## 1. Introduction

Today, membrane technologies occupy one of the most intensively developing technological areas and find various applications in industry [1]. A striking example is the membrane contactor, which allows for the implementation of the separation process or a chemical transformation. In the membrane contactor, the membrane acts as an interface between two phases [2]. The main advantages of membrane contactors are independent phase flow rates within a wide range without mutual dispersion of phases and large specific mass-transfer areas per module volume—up to 10,000 m^2^/m^3^. Moreover, they lead to increased mass-transfer coefficients of the required component from one phase to another and, consequently, to compact separation modules [3]. Gas–liquid membrane contactors are already widely used for various applications, including blood oxygenation [4,5], the removal of acid gases (CO_2_, H_2_S, SO_2_) from gaseous media [6,7], the removal of dissolved gases from liquids [8,9], and the separation of saturated and unsaturated hydrocarbons [10]. Many reviews of recent years [3,11,12,13,14] are devoted to membranes for gas–liquid membrane contactors and their application areas.

Liquid–liquid membrane contactors are the second critical application, where the mass transfer between two liquid phases is realized, while the phases can be both miscible and immiscible. In the first case, contact between miscible liquids on a porous membrane is realized, where the mass transfer of the target component is carried out in the gas-filled membrane pores. This is the case for several membrane distillation configurations [15], which have emerged as an independent field of membrane science. Other examples are the removal of ammonia from wastewater by sulfuric acid solutions [16,17], the delivery of dissolved carbon dioxide into the growth medium of micro-organism cultures (algae and cyanobacteria) [18], and juice concentration [19].

In the second case, two immiscible liquid phases (mainly aqueous and organic) are in contact on porous membranes. Therefore, this case attracts a lot of attention since the advantages of membrane contactors can be translated into the extraction of various substances [20]. The foundations of liquid–liquid membrane extraction (LLME) were built in the mid-1980s by the pioneering work of the K. Sirkar [21] and E.L. Cussler [22] groups. Today, its application is highly diverse and includes the separation of metals [23] from wastewater, the removal of organic acids, alcohols, and ketones from fermentation broths [24,25], and the extraction of various pharmaceuticals, hormones [26], and antibiotics [27] from aqueous solutions. Moreover, this method could be used to solve various petrochemical problems. For example, as shown recently, it is possible to separate sulfur-containing components from oil [28] and aviation fuels [29]. At the same time, liquid–liquid membrane contactors can be applied to purify biodiesel fuel from unreacted components (rapeseed oil, methanol, glycerin) [30].

In membrane liquid–liquid contactors–extractors, the transfer of the target (most often non-volatile) component occurs during direct contact of the phases at the mouths or inside the membrane’s pores. As in the case of gas–liquid contactors, the stability and controllability of the mass-transfer interface in the membrane pores (indicated by the absence of mutual dispersion of phases) are the keys to the efficient operation and scalability of the separation equipment. Special features of the phase contact depend on the technological parameters of the membrane-extraction system (linear phase flow rates, the pressure drop between phases, liquid–liquid interfacial tension, the process duration). They also depend on membrane functional parameters (thickness, pore size and geometry, structural asymmetry, hydrophilicity/hydrophobicity of the pore surface, and affinity for contacting liquids). Some of these parameters were studied in the early works of Professor Sirkar’s group. For example, in [31], the authors revealed the effects of the pore size and tortuosity of polypropylene (PP) and polytetrafluoroethylene (PTFE) membranes in combination with varying the liquid velocity and pressure drop. In [32,33], the effect of the hydrophilicity/hydrophobicity of the membrane material on the process parameters was evaluated. The authors used regenerated cellulose/cellulose acetate (hydrophilic), PP, and PTFE (hydrophobic) membranes. Nevertheless, the use of membranes made of various materials for this kind of research suffers from a significant drawback.

The material plays a primary role in the organization of the pore structure of the membrane, from which a logical consequence follows: membranes made of different materials have an incomparable porous structure. For example, commercial hydrophobic PP and PTFE membranes are obtained mainly by extrusion of a polymer melt followed by stretching, which leads to the formation of symmetric slit pores [34,35]. On the other hand, hydrophilic porous membranes, including cellulose derivatives, are obtained by a more common phase inversion technique using nonsolvents [36,37]. In this process, a symmetric/asymmetric spongy and/or finger-like pore structure of the membrane is obtained. As a result, conclusions about the relationship between the functional properties of the membrane’s porous structure and the technological parameters of the membrane contactor system are drawn based on a comparison of membranes with incomparable porous structures and may be incomplete.

To fill this gap, in this work, for the first time, asymmetric track-etched membranes made of polyethylene terephthalate (PET) were investigated in the mode of a liquid–liquid membrane contactor.

Among the asymmetric membranes, those produced using the track-etching method possess a number of special properties. The track-etching technique provides the possibility of fabricating membranes with precisely determined structural characteristics, including thickness, number of pores, and pore geometry (shape, orientation, symmetry/asymmetry) [38]. Track-etched membranes have a large number of applications. For example, hydrophobized track-etched membranes have much potential in membrane distillation. Due to their properties, such membranes can be used as model membranes for the development and confirmation of theoretical mass, heat transfer, liquid entry pressure, and fouling. Track-etched membranes also have a wide range of applications in the ultrafiltration and microfiltration of liquids and gases; in the analytical control of substances; and in the food and pharmaceutical industries, microelectronics, and other areas of science and industry [39]. Both symmetric and asymmetric small-pore track-etched membranes are widely employed to study the ionic transport phenomena in nanoscale volumes, to build nanofluidic devices, such as molecular sensors, pumps and gates, and logical elements [38], and to develop various new processes, such as electro-baromembrane ion separation [40]. Thus, in [41], a hybrid electro-baromembrane process was applied to separate monovalent cations (K^+^/rhodamine and K^+^/Li^+^). For that, a track-etched membrane with 40-nm pores produced from PET was used. This is an example of a fundamentally new application of small-pore track-etched membranes. Selective K^+^/Mg^2+^ separation was performed on track-etched polycarbonate membranes with 30-nm pores modified by adsorption of poly(styrene sulfonate) (PSS)/protonated poly(allylamine) (PAH) films [42] The study [43] concerns Li^+^/K^+^ separation by using polycarbonate track-etched membranes containing cylindrical pores with nominal 30-nm or 10-nm diameters. Such pores are small enough to provide strong anion exclusion throughout the pore at low ionic strength. This exclusion leads to large streaming potentials and high Li^+^/K^+^ selectivities. At the same time, the pores are large enough to allow sufficiently high flow rates under readily available transmembrane pressures.

The performance of asymmetric nanopores in a specific application critically depends on the pore geometry, especially on the shape and size of the narrow part of the pore. The asymmetric track-etched membranes can be produced using either one-sided (also referred to as asymmetric) etching [44] or surfactant-controlled etching [45] of ion tracks in a polymer foil. Both methods allow for the fabrication of highly asymmetric channels with openings on the nanometer scale at one end and 1–2 orders of magnitude larger openings at the other end. The former method is employed mostly in the case where single pores should be produced. The latter is more practical when multi-pore membranes are needed [45]. Using the advantages offered by this technology, we fabricated and employed in the present work a series of membranes of the same thickness, but with different sizes of highly asymmetric pores and with a small increment in the pore diameter on the selective side.

To study the stability of the interface in a membrane contactor, two systems were used that significantly (by an order of magnitude) differ in the value of the interfacial tension.

## 2. Materials and Methods

### 2.1. Materials

Distilled water with a specific conductance of less than 6 μSm/cm was used as the model aqueous phase. Hexadecane (98%) and 1-pentanol (99%) (Component Reaktiv, Moscow, Russia) were used as model organic phases. Their properties are presented in Table 1. As one can see, the properties of 1-pentanol and hexadecane are pretty similar except for the interphase tension with water. In this case, the value for “water–1-pentanol” is by an order of magnitude lower than that for the “water–hexadecane” system.

Distilled water and diiodomethane (99%, Sigma-Aldrich, Schnelldorf, Germany) were used to evaluate the surface properties of the membranes. In addition, acetone (99.9%, Chimmed, Moscow, Russia), distilled water, and hexadecane were used to investigate the effect of an asymmetric membrane’s arrangement in the model mass transfer process from an organic phase to an aqueous phase. All the chemicals were used without further purification.

### 2.2. Track-Etched Membranes

PET membrane samples with pores of controlled geometry were obtained by the ion-track method. The pore system in these membranes is formed by irradiating a Hostaphan RNK film with a nominal thickness of 23 ± 1 μm with the 210 MeV krypton ions from the U-400 cyclotron of the Flerov Laboratory of Nuclear Reactions, JINR. The polymer film with a width of 31 cm was transported across the scanned ion beam. The width of the irradiated area was 21 cm. In order to minimize the effects of overlapping adjacent pores, the angles of entry of ions into the film were distributed in the interval ±30° to the surface normal.

Samples of A4 size were cut from the film and exposed to ultraviolet radiation with a power of ~4 W/m^2^ in the range of 280–315 nm on one side for 12 h to impart asymmetry. Chemical etching was carried out in a 3 M NaOH solution with 0.0125% Dowfax 2A1 surfactant at 60 °C [45]. As a result, channels were formed with a diameter varying across the film thickness.

Small pieces were cut from the fabricated membrane samples and used for testing using SEM and other methods. The rest were employed in the experiments on extraction in the liquid–liquid contactor. The volume porosity of the membranes was determined by the formula:

(1) $$\Pi =100\%\cdot \frac{m_{un}-m_{ir}}{m_{un}}$$

where $m_{un}$ and $m_{ir}$ are the masses of samples cut from unirradiated and irradiated areas, respectively. The sample size was 4 × 4 cm. The accuracy of weight measurements was 0.00001 g.

The thickness of the membranes was measured using a Mitutoyo Litematic VL-50 instrument (accuracy: ±0.1 μm).

### 2.3. Membrane Characterization

#### 2.3.1. Scanning Electron Microscopy

The porous structure of the membranes was investigated by scanning electron microscopy (SEM). SEM was performed using the SU8020 microscope with a cold cathode (Hitachi, Japan). In order to improve the resolution and contrast of images, a thin layer of the gold–palladium alloy was deposited on the samples. For the determination of the surface pore diameter, all SEM images were recorded under identical conditions. The transverse size of pore openings was measured using the GATAN DIGITAL MICROGRAPH software. For each sample, 50–80 pores were analyzed. Cross-sections of the membranes were prepared after embrittlement with UV for about 100 h in air [47].

#### 2.3.2. Surface Properties of the Membranes

The surface properties of the membranes were investigated by determining the contact angles for water and diiodomethane. The measurements were carried out on both sides of the track-etched membranes at room temperature (23 ± 2 °C).

Contact angle values were measured via the conventional sessile drop technique using the LK-1 goniometer. Each value was determined as a mean for five measurements. For image capture and digital processing of the drop images, the DropShape-software-provided Laplace–Young contact angle calculation was used. The measurement error was 2°.

The membrane surface energy value was determined according to the Owens–Wendt method [48]. The technique allows for the calculation of the surface energy value *γ* as a sum of polar *γ^p^* and *γ^d^* dispersive components using two different liquids. The relation between the surface energy and the equilibrium contact angle of the liquid phase placed onto the solid phase is derived from the Fowkes equation [49]:

(2) $$\gamma_{l}\left(1+cos\theta \right)=2{\left(\gamma_{l}^{d}\gamma_{s}^{d}\right)}^{1/2}+2{\left(\gamma_{l}^{p}\gamma_{s}^{p}\right)}^{1/2}$$

where the superscripts «*d*» and «*p*» relate to the dispersive and polar components of the liquid surface energy (*γ_l_*) and the membrane surface (*γ_s_*), respectively.

Water and diiodomethane were used as test liquids, as the surface energy components of both liquids are well known and widely described in the literature [50,51].

#### 2.3.3. Dynamic Light Scattering

The presence or absence of the mutual dispersion of liquids was controlled by the dynamic light scattering (DLS) method. For this, a 1-mL sample of each liquid was taken at the end of the hour-long experiment for study using a Malvern Zetasizer Nano ZS dynamic light scattering analyzer.

### 2.4. Liquid–Liquid Membrane Contactor

**Figure 1:**
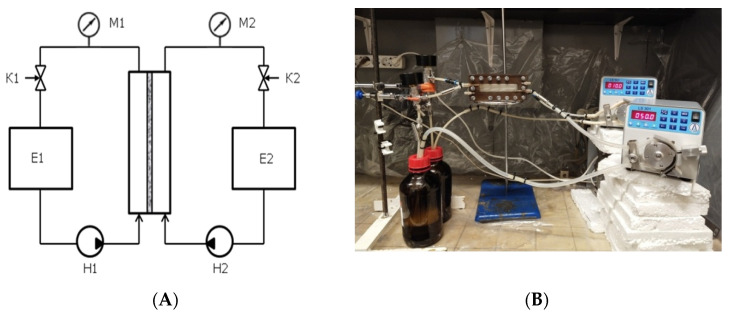
(**A**) Structural scheme and (**B**) external view of the setup for determining the stability of the interface in the liquid–liquid membrane contactor. M1, M2—manometers; K1, K2—fine adjustment valves; E1, E2—technological reservoirs containing water and organic liquid, respectively; H1, H2—peristaltic pumps.

To study the mass transfer interface’s stability during the operation of a liquid–liquid membrane contactor, a piece of experimental equipment with a flat-sheet membrane module was fabricated. The structural scheme and an external view of the piece of equipment are shown in Figure 1.

The main working element was a flat-sheet membrane module whose liquid channels had a rectangular geometry. The active area of the membrane was 21.2 cm^2^. The vertical arrangement of the contactor avoids the formation of gas bubbles and stagnant zones during the liquid flow in the module’s internal channels. The supply of liquids to the membrane contactor was carried out in the counter-current flow mode. The equipment allows for the variation and control of the liquid velocities and differences in their pressures. In order to study the presence or absence of mutual dispersion in the membrane contactor for a water–organic liquid pair, 1-pentanol and hexadecane with different surface energies and interfacial tensions of the water–organic systems were selected. Unless otherwise specified, the following mode was used in the work: the linear velocities of aqueous and organic phases are 2.4 and 0.5 cm/s, respectively; the organic liquid is at atmospheric pressure; and the water overpressure is 3 kPa.

In addition, studies were carried out to separate a small amount of pollutant from the organic liquid into the aqueous phase using a liquid–liquid membrane contactor with a different arrangement of the asymmetric track-etched membrane. For this purpose, a 1% solution of acetone in hexadecane was used.

The concentration of solutions was determined using a Crystallux-4000M gas chromatograph. The chromatograph was equipped with an autosampler and a flame ionization detector. We used a Phenomenex Zebron ZB-FFAP capillary column (length, 50 m; diameter, 0.32 mm; phase thickness, 0.50 μm). The data evaluation was done with NetChrom software.

## 3. Results and Discussion

### 3.1. Track-Etched Membranes

**Figure 2:**
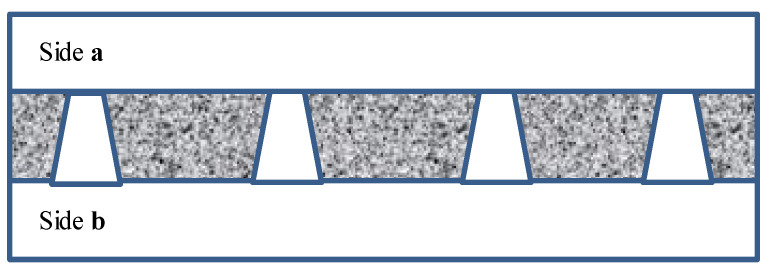
Schematic representation of the cross-section of asymmetric membranes.

Figure 2 schematically shows a track-etched membrane with an asymmetric pore structure. For convenience, we designate the side of the track-etched membrane with a smaller diameter of the asymmetric pore as side **a**, and the side with the larger pore diameter, respectively, as side **b**.

#### 3.1.1. Characterization of Track-Etched Membranes Using SEM

**Figure 3:**
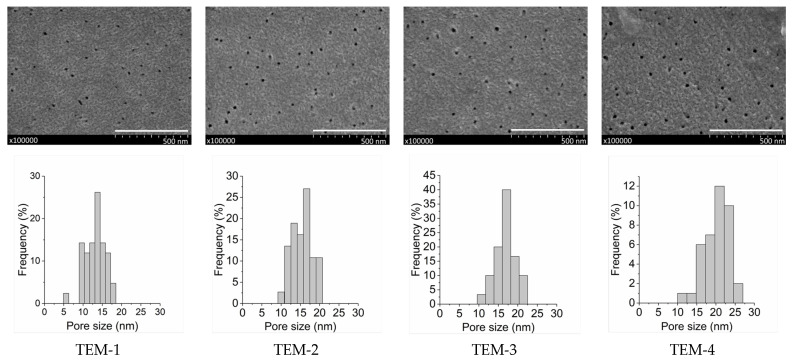
SEM images (**top**) and pore size distributions (**bottom**) of side **a** of membranes TEM-1, TEM-2, TEM-3 and TEM-4 (from the left to the right). Scale bar, 0.5 μm.

**Figure 4:**
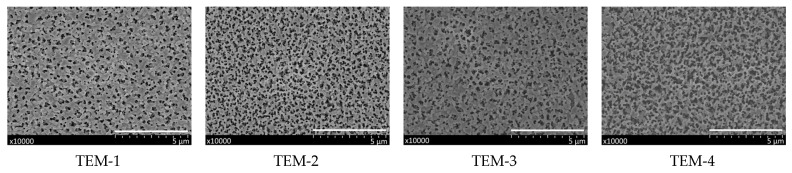
SEM images of side **b** of membranes TEM-1, TEM-2, TEM-3 and TEM-4 (from the left to the right). Scale bar, 5 μm.

The porous structure of the track-etched membranes was investigated by scanning electron microscopy. SEM images of sides **a** and **b** for four studied membranes are presented in Figure 3 and Figure 4. The distributions of pore sizes for side **a** evaluated by using the GATAN software can also be seen in Figure 3.

**Figure 5:**
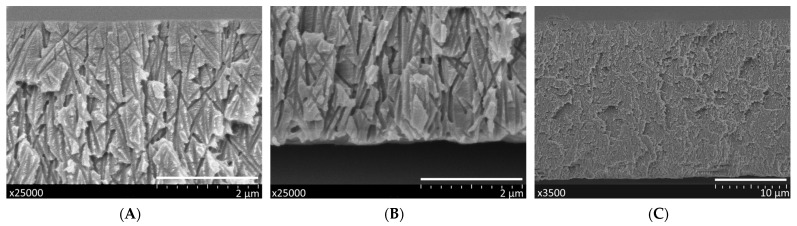
SEM images of a typical cross-section of TEM-1: the layers adjacent to sides **a** and **b** (images (**A**) and (**B**), respectively) and full thickness (**C**). Scale bars are shown in the frames.

Figure 5 shows a typical cross-section of the asymmetric track-etched membrane with high porosity, using the example of TEM-1. The top layer, the bottom layer, and a full cross-section are shown.

Depending on the etching time, the average pore diameter on side **a** varies in the range of 12–19 nm. On the other side of the membrane, the entrances of individual pores merge and form a sponge-like structure because the apparent porosity is close to 100%. The pores consist of a short funnel-shaped entrance on the UV-treated side, a long cylindrical part, and a short (ca. 1-μm-long) conical tip. This configuration is typical of the pores produced by the procedure used (see, for instance, Figure 4 in [52], where images of individual pore channels are presented). A closer look at the SEM photos in Figure 5 also confirms the above geometry.

Table 2 presents the structural characteristics of the studied membranes. It is important to emphasize that all four membranes possess practically the same thickness (23 ± 1 μm), have the same pore density (4.5 × 10^9^ cm^−2^), and differ only in the pore size. It should be noted that commercial films do not have a constant thickness. The thickness of 23 microns is the nominal value. In reality, the thickness varies along and across the film in the roll at least within the range of 23 ± 1 mm, and sometimes even beyond this range. Moreover, the reader should take into account that we did not measure the thickness over the whole sample because the sample should have been clean and should not have been damaged by the gauge probe as a result of being excessively manipulated before use in the contactor. In fact, the deviations in the membrane’s thickness did not actually have an observable influence on the membrane contactor system’s performance. The average pore diameters on the selective side increased from approximately 12 nm to 19 nm. These values are arbitrary to a certain extent because SEM cannot provide sufficiently accurate data in this range of dimensions. However, the sample size was large enough to make sure that the data confirmed a regular increase in the surface diameter with increasing etching time. All the SEM photos of membrane surfaces were taken under identical conditions, and, therefore, in the case of a bias, the bias would be similar for all four membrane samples.

#### 3.1.2. Surface Properties of the Membranes

**Figure 6:**
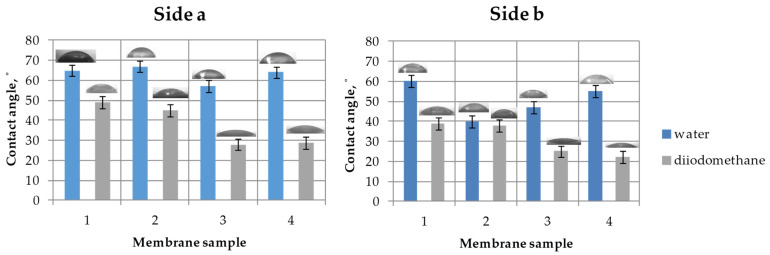
Water and diiodomethane contact angles: **left**—side **a**; **right**—side **b**.

Figure 6 shows the results of measuring the water and diiodomethane contact angles for the studied membranes. All samples of track-etched membranes are moderately hydrophilic with water wetting angles varying from 40° to 67°. It is also possible to note the difference in the contact angles of different sides of the asymmetric membranes. For all the membranes, side **b**, which was exposed to UV irradiation before etching, has increased hydrophilicity (a decrease in the water contact angle value).

The results of calculating the surface energy of the membranes are presented in Table 3. An increase in the hydrophilicity of side **b** with larger pores compared with side **a** with smaller pores is also expressed in the increase in the polar component of the surface energy, *γ^p^*. Consequently, the total value of the surface energy also increases. It should be noted that the highest value of the polar component of the surface energy (*γ^p^* = 31 mJ/m^2^) was obtained for side **b** of TEM-2. As a result, side **b** of TEM-2 has the highest surface energy of 59 mJ/m^2^. A similar nonlinear effect of the sodium hydroxide etching procedure on the roughness and surface properties of the membranes was observed in [53].

The pentanol and hexadecane contact angles of the membranes were also measured. It was found that both sides of all membrane samples were thoroughly wetted with pentanol and hexadecane (the contact angles were less than 10°).

From the data on wettability, it can be concluded that the porous structure of the membranes will be filled with organic liquid when the membranes come into contact with aqueous and organic phases. In this regard, it is logical to maintain an overpressure of water when the membrane contactor is in operation.

### 3.2. Liquid–Liquid Membrane Contactor System

#### 3.2.1. Influence of the Porous Structure of the Membrane on the Stability of the Phase Contact Interface

Table 4 shows the effect of the arrangement of the sides of the track-etched membranes in the “water–pentanol” and “water–hexadecane” systems under the following conditions: linear velocity of water = 2.4 cm/s; linear velocity of organic liquid = 0.5 cm/s; ΔP = 3 kPa.

For the “water–pentanol” pair, there is no stabilization of the interface for all studied membranes in the case of water supply from side **a**. It should be noted that this system is characterized by low interfacial tension (4.5 mN/m). With a change in the membrane arrangement in this system, the water supply from side b leads to the stabilization of the contact surface of the phases for TEM-1 and TEM-2 with pore sizes of 12.5 and 14.7 nm, respectively. However, for TEM-3 and TEM-4, which have larger pore diameters, the stability of the interface is still absent, and mutual mixing of phases was observed.

A somewhat different picture was observed for the “water–hexadecane” system with high interfacial tension (55.2 mN/m). The liquid–liquid interface for TEM-1 and TEM-2 with pore sizes of 12.5 and 14.7 nm, respectively, is stable regardless of the membrane arrangement towards water or hexadecane. However, for TEM-3 and TEM-4, which have larger pore sizes (15.7 and 19.0 nm, respectively), the stability of the interface was not observed, as in the case of the “water–pentanol” system.

Thus, a preliminary conclusion can be drawn that the water supply from side **b** with the larger asymmetric pore size is preferable for the stable operation of the liquid–liquid membrane contactor, regardless of the considered water–organic system. In addition, it can be concluded that the pore size of the TEM-3 and TEM-4 samples exceeds the required diameter (approximately 15 nm) for the implementation of stable operation of the contactor. In this regard, TEM-1 and TEM-2 were further investigated.

From our point of view, this behavior can be explained by the existence of a threshold value for a specific combination of membrane pore size and liquid–liquid interfacial tension. This value is higher than that obtained for TEM-2 but lower than that obtained for TEM-3. The exceedance of this value in the TEM-3 case and in the TEM-4 case results in the presence of mutual dispersion of the “water–hexadecane” pair as stated in Table 4. The second possible explanation for this case is the difference in the surface energy of the studied membranes.

#### 3.2.2. Influence of a Drop in Pressure between Phases on the Interphase Stability

Excessive water pressure is necessary to control the organic phase’s flow through the membrane’s pores, which thoroughly wets both its surfaces. Table 5 shows the results of the experiment on the interphase stability for the “water–hexadecane” system within contactors based on TEM-1 and TEM-2. The excessive water pressure was varied within the range of 3–13 kPa. The following two conclusions follow from the data shown in Table 5. First, the stability of the contactor is higher for both studied membranes in the case of water supply from side **b**, that is, from the side whose asymmetric pores have a larger diameter. This result confirms the conclusion drawn in the previous section. Second, TEM-2 is more stable than TEM-1. This result contradicts the fact that the pore diameter of TEM-2 (14.7 nm) is larger than the pore diameter of TEM-1 (12.5 nm). The highest value of the polar component of the surface energy (31 mJ/m^2^) and the value of the surface energy (59 mJ/m^2^) of side **b** (Table 3) may be possible explanations for the increased stability of TEM-2.

#### 3.2.3. Influence of the Parameters of the Membrane-Contactor System on the Stability of the Liquid–Liquid Interface

The results of varying the liquid velocities on the mutual dispersion of phases for TEM-2 and the “water–hexadecane” system are presented in Table 6. In all cases, ∆P = 3 kPa. TEM-2 and the “water–hexadecane” system were chosen because they were found to be the most stable.

As can be seen, the stability of the liquid–liquid interface is more sensitive to variations in the water’s velocity than to variations in the organic liquid’s velocity. As shown in [54], the system’s resistance caused by the water flow is the determining factor. When the water’s velocity increases to a specific limit, the shear force of the water phase flow prevails over the interfacial tension, and the drops begin to detach. Additionally, the drop size decreases with an increase in the water’s velocity, which also agrees with the data given in [54].

#### 3.2.4. Discussion of Obtained Results

**Figure 7:**
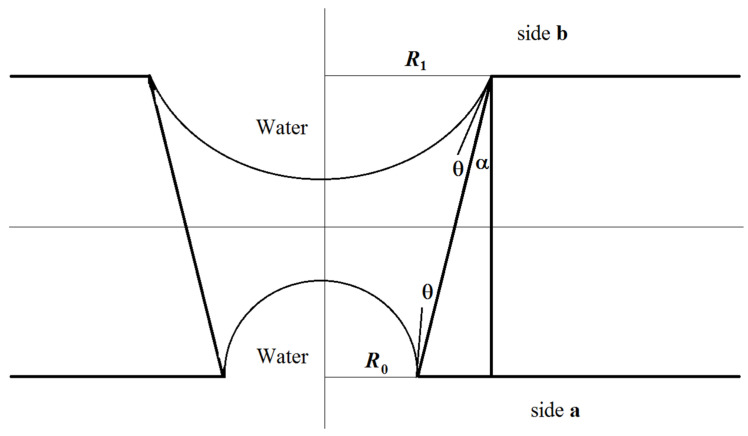
Scheme of the cone channel and possible profiles of the menisci of the organic component at the boundary with water.

Let us consider the formation of a meniscus in a water–membrane–organic system. Figure 7 shows the scheme of the conical channel and possible profiles of menisci of the organic component at the boundary with water in the case of water supply from the side with larger and smaller channel radii in the equilibrium state, subject to compensation for capillary pressure. Here, θ is the contact angle, α is the cone angle, R_0_ is the radius of the channel of side **a** (see Table 2), and R_1_ is the radius of the channel of side **b**. Estimates show that the angle value is several degrees for the characteristic radius R_1_ on the order of several nanometers and for a membrane thickness of 23 μm (see Table 2). The breakdown and penetration of organic particles into the water when the water’s location is on side **a** or side **b** will probably occur after an increase in pressure from the organic side, leading to the disappearance of the meniscus. A further increase in pressure can lead to the formation of a meniscus above the membrane from the water side and the separation of organic particles by the water’s flow.

**Figure 8:**
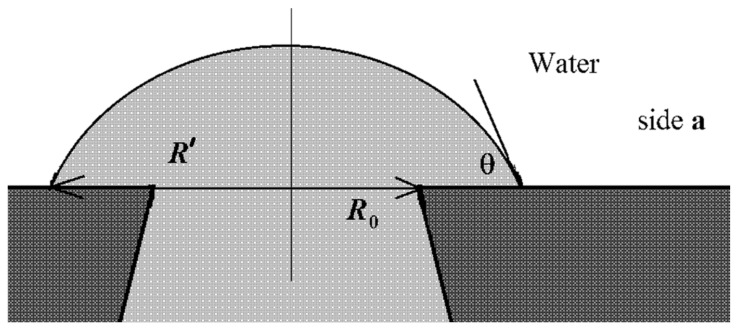
Scheme of the interaction of the water–insoluble organic liquid with the surface of the material and water on side **a**.

However, for the case with water on side **a**, the value $\Delta p\sim \frac{2\gamma \cos\theta }{R_{0}}$ will be on the order of tens of atmospheres and cannot be compensated for by water pressure on side **a**. The most probable scenario for developing the interaction of the water–insoluble organic liquid with the surface of the material and water is shown in Figure 8.

The organic liquid partially spreads over the membrane’s surface until an equilibrium is established between the energy of formation of the “organic component–membrane” surface and the energy of formation of the “water–organic component” surface:

(3) $$\gamma_{mo}\Delta S_{mo}=\gamma_{wo}\Delta S_{wo}$$

where $\gamma_{mo}$ is the energy of formation of the “membrane–organic liquid” surface, $\Delta S_{mo}$ is the surface area of the contact between the membrane and the organic liquid, $\gamma_{wo}$ is the energy of formation of the “water–organic liquid” surface, and $\Delta S_{wo}$ is the surface area of the contact between the water and the organic liquid. Thus, the membrane surface on side **a** will already contain an insoluble organic liquid, which explains the lower transmembrane pressure required to detach this component from the surface and, accordingly, the lower water velocity.

**Figure 9:**
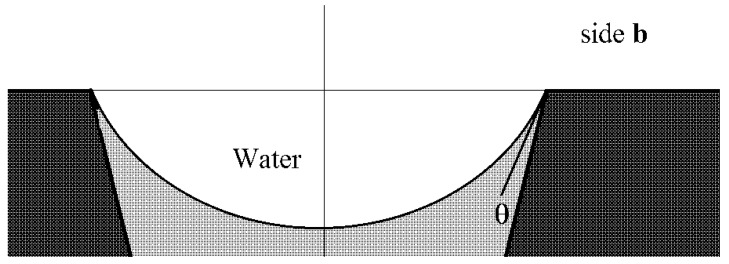
Scheme of the interaction of the water–insoluble organic liquid with the surface of the material and water on side **b**.

In the case where the water phase’s location is on side **b**, the rise of the organic liquid due to the formation of an energetically favorable “organic liquid–membrane” surface will be compensated for by an increase in the energetically unfavorable “water–organic liquid” surface (3). In this case, the scheme shown in Figure 9 will most likely be implemented.

It should be pointed out that the surface of the membrane on side **b** has a crumbling, spongy structure (see Figure 4). Consequently, the near-surface layer of the membrane will predominantly contain water rather than the organic component.

Within the framework of such physical concepts, it is also possible to explain the difference in results for different organic components (hexadecane and 1-pentanol). In the case of 1-pentanol, the value $\gamma_{wo}$ is several times lower than that for hexadecane (see Table 1). This means that the formation of an energetically unfavorable “water–organic liquid” surface in this case limits the spread of the organic component over the membrane surface to a lesser extent and, therefore, the probability of the organic liquid’s penetration into the water increases.

In practice, the obtained track-etched membranes might be promising, e.g., for a process such as membrane emulsification (for example, for pharmaceuticals). This is a process in which two immiscible phases are brought into contact in a membrane module, while one phase is forced into the other through the membrane pores, and as a result an emulsion is formed in the second phase. Obviously, in this case, one of the primary roles in the resulting properties of the derived emulsion is played by the membrane’s porous structure. The more monomodal the pore distribution, the narrower the size distribution of the emulsion drops. The lower the concentration and the more uniform the distribution of pores on the surface, the lower the probability that drops will merge at the mouths of two adjacent pores, and, again, the narrower the drop size distribution (see, for example, [55,56]).

### 3.3. Extraction of Acetone from the Organic Phase to the Aqueous Phase in the Membrane Contactor

**Figure 10:**
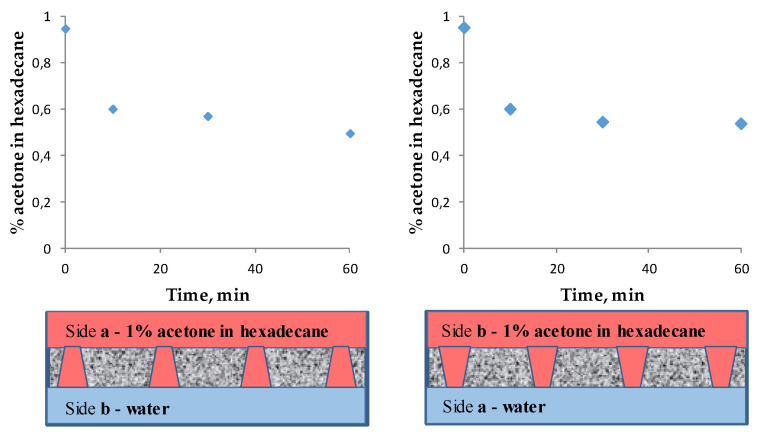
Decrease in the acetone content in the organic phase in the liquid–liquid membrane contactor with different membrane arrangements.

Experiments on the extraction of a small amount of pollutant from the organic phase to water using the developed liquid–liquid membrane contactor with different arrangements of the asymmetric track-etched membrane were carried out. It is known that oxygenates, such as carboxylic acids, aldehydes/ketones, and alcohols, are often present in less than 10 wt.% concentrations in the apolar organic solvents from which they are to be recovered [57,58]. In this work, the extraction of acetone from its 1% solution in hexadecane to the aqueous phase was tested in the developed liquid–liquid membrane contactor using the asymmetric membrane TEM-1. Figure 10 shows a decrease in the acetone content in the organic phase of up to 50% regarding the initial concentration. However, the arrangement of the membrane’s sides does not actually affect the efficiency of the transfer. Therefore, it is better to realize the mass transfer process in the membrane contactor extraction system with the water at side **a**. This case allows us to apply a larger pressure difference between the phases at the same efficiencies of target component extraction.

Thus, the liquid–liquid contactor based on track-etched membranes distinctly allows for the extraction of unwanted contaminants from the organic phase to the aqueous phase.

## 4. Conclusions

In this work, track-etched PET membranes with highly asymmetric pores were prepared. The pore sizes varied from 12.5 nm to 19 nm, while the values on the other side of the membrane were an order of magnitude higher. The data on the surface properties indicate that the membranes are lyophilic, i.e., wetted with both water and organic phases. The obtained membranes demonstrate surface energy values in the range of 41–59 mJ/m^2^. It was established that the asymmetry of the porous structure has an influence on the tolerable parameters of liquid–liquid membrane contactor. For example, water supply from the side with a larger pore size is preferable for the stable operation of the liquid–liquid contactor. Additionally, in the case of asymmetric membranes it is possible to expand the range of values of the drop in pressure between the phases by at least two times (from 5 to 10 kPa), which does not lead to mutual dispersion of the liquids, while in symmetric membranes, a strict value of the drop in pressure should be maintained.

The stability of the liquid–liquid interface increases with decreasing pore size. At the same time, the organization of the process in which water is supplied on the side with larger pore sizes is preferable for implementing a membrane contactor system since it leads to a more stable liquid–liquid interface, particularly for a system with lower interfacial tension.

The stability of the liquid–liquid interface in the membrane contactor is more sensitive to variations in the water velocity than to variations in the organic liquid velocity. In this case, with an increase in the water flow rate, the drop size decreases.

Finally, we have demonstrated that the liquid–liquid contactor based on the fabricated track-etched membranes makes it possible to efficiently extract pollutants from the organic phase into the aqueous phase using a 1% solution of acetone in hexadecane as a model system.

## Figures and Tables

**Table 1:** Properties of liquids used for the membrane contactor [46].

Property	Aqueous Phase	Organic Phase	
	Water	Hexadecane	1-Pentanol
Molar mass, g/mole	18	226.45	88.15
Normal boiling point, °C	100	286.8	137.9
Viscosity at 25 °C, mPa·s	0.895	3.08	3.36
Surface tension at 25 °C, mN/m	71.98	27.15	25.3
Interfacial tension of the water–liquid interface at 25 °C, mN/m	-	55.2 ^1^	4.5

^1^ The value is presented at 22 °C.

**Table 2:** Structural characteristics of track-etched membranes.

Sample	Time of Chemical Etching, min	Average Pore Diameter on the Selective Side Determined by SEM, nm	Thickness, μm	Volume Porosity, %
TEM-1	3	12.5 ± 0.3 *	23.7	14
TEM-2	3.5	14.7 ± 0.4	22.8	17
TEM-3	4	15.7 ± 0.5	22.7	22
TEM-4	4.5	19.0 ± 0.5	22.6	32

* Standard deviation of the mean.

**Table 3:** The surface energy of the membranes.

Sample	Surface Energy, mJ/m^2^					
	Side a			Side b		
	*γ^d^*	*γ^p^*	*γ*	*γ^d^*	*γ^p^*	*γ*
TEM-1	26	15	41	30	17	47
TEM-2	28	13	41	28	31	59
TEM-3	35	16	51	34	23	57
TEM-4	35	12	47	36	17	53

*γ^d^* is the polar component and *γ^p^* is the dispersive component of the surface energy *γ*.

**Table 4:** Influence of the arrangement of the sides of the track-etched membranes.

Sample	Interphase Surface Stability			
	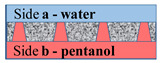	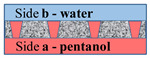	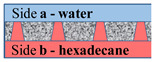	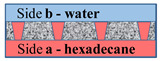
TEM-1	-	+	+	+
TEM-2	-	+	+	+
TEM-3	-	-	-	-
TEM-4	-	-	-	-

**Table 5:** Influence of water overpressure on the interphase stability of the “water–hexadecane” system for TEM-1 and TEM-2.

Water Overpressure, kPa	Interphase Surface Stability			
	TEM-1		TEM-2	
	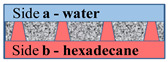	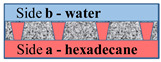	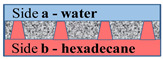	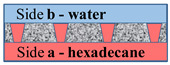
3	+	+	+	+
5	-	+	+	+
7	-	+	-	+
10	n/a	-	-	+
13	n/a	n/a	n/a	-

**Table 6:** Effect of operational parameters of the membrane contactor on the liquid–liquid interface stability for TEM-2.

Side a—Hexadecane Side b—Water				Side a—Water Side b—Hexadecane			
The linear velocity of water = 2.4 cm/s; the linear velocity of hexadecane varies		The linear velocity of water varies; the linear velocity of hexadecane = 2.6 cm/s		The linear velocity of water = 2.4 cm/s; the linear velocity of hexadecane varies		The linear velocity of water varies; the linear velocity of hexadecane = 2.6 cm/s	
The linear velocity of hexadecane	DLS	The linear velocity of hexadecane	DLS	The linear velocity of hexadecane	DLS	The linear velocity of hexadecane	DLS
1.6	-	1.6	-	1.6	-	1.6	-
1.9	-	1.9	-	1.9	-	1.9	-
3	-	3	-	3	-	3	-
3.3	-	3.3	-	3.3	-	3.3	-
3.9	hexadecane drops (176 nm) in water	3.9	hexadecane drops (176 nm) in water	3.9	hexadecane drops (176 nm) in water	3.9	hexadecane drops (176 nm) in water

## Data Availability

The data presented in this study are available on request from the corresponding author.
